# The Effect of Rice vs. Wheat Ingestion on Postprandial Gastroesophageal Reflux (GER) Symptoms in Patients with Overlapping GERD-Irritable Bowel Syndrome (IBS)

**DOI:** 10.3390/foods11010026

**Published:** 2021-12-23

**Authors:** Tanisa Patcharatrakul, Sittikorn Linlawan, Suppawatsa Plaidum, Sutep Gonlachanvit

**Affiliations:** 1 Division of Gastroenterology, Department of Medicine, King Chulalongkorn Memorial Hospital, The Thai Red Cross Society, Bangkok 10330, Thailand; dr_tanisa@yahoo.com (T.P.); vavai_vv@hotmail.com (S.P.); 2 Center of Excellence on Neurogastroenterology and Motility, Faculty of Medicine, Chulalongkorn University, Bangkok 10330, Thailand; 3 Department of Medicine, Phrachomklao Hospital, Phetchaburi 76000, Thailand; sittilin@yahoo.com

**Keywords:** rice, wheat, fermentable oligosaccharides, disaccharides, monosaccharides, and polyols (FODMAPs), irritable bowel syndrome, GERD, intestinal gas, fermentation

## Abstract

A randomized crossover study in twenty-one patients (18F, age 50 ± 13 years) with overlapping GERD-IBS was conducted to evaluate the effects of rice noodles (low FODMAPs) vs. wheat noodles (high FODMAPs) on typical GER symptoms, and the correlation between GERD symptoms and intestinal gas production. Results: Heartburn and regurgitation scores were highest in most patients (19/21) during the first 15 min after meals. At 15 min after lunch, wheat was significantly associated with more regurgitation and heartburn than rice. Also, at 15 min after breakfast, wheat aggravated more regurgitation than rice. Wheat ingestion was significantly associated with higher H_2_ and CH_4_ levels after lunch compared to rice, whereas gas levels before lunch were similar (*p* > 0.05). The area under the curve of H_2_ and CH_4_ concentration 15 min after a lunch of wheat moderately correlated with the regurgitation severity at 15-min (r = 0.56, *p* < 0.05). Conclusion: Wheat induced more GERD symptoms than rice in patients with overlapping GERD-IBS. This effect, immediately developed after lunch, was associated with more intestinal gas production. Thus, a low FODMAPs diet may relieve postprandial GERD symptoms in GERD patients with overlapping IBS. Wheat inducing more regurgitation than rice after breakfast suggests other mechanism(s) besides gut fermentation.

## 1. Introduction

Food can exacerbate gastrointestinal (GI) symptoms in patients with functional gastrointestinal disorders as a consequence of several mechanisms, such as changes in intestinal motility, visceral sensations, microbiome, intestinal permeability, immune activation, and brain–gut interactions [1,2]. Carbohydrates, a major component of each meal, may cause bloating, flatulence, abdominal pain and discomfort, and diarrhea if the small intestine′s absorption process is not complete. The products of bacterial fermentation in the colon, such as hydrogen (H_2_), methane (CH_4_), and short-chain fatty acids (SCFAs), can modulate colonic propagated contraction, and colonic transit [3,4]. Studies showed that colonic infusion of SCFAs affects upper GI tract motor functions, including relaxation of the proximal stomach [5], and induces transient lower esophageal sphincter relaxations (TLESRs) [6] in healthy individuals. Research in animals also suggested the role of SCFAs on gut–brain communication and brain function directly or indirectly through immune, endocrine, vagal, and other humoral pathways [7].

Overlapping functional gastrointestinal disorders are common, and associated with poor treatment outcomes and lower quality of life [8]. IBS and GERD have been reported in about one-third of patients with each disease [9,10]. Meta-analysis of randomized controlled studies supported the benefit of consuming a low FODMAPs diet on gastrointestinal symptoms and quality of life in patients with IBS [11,12]. However, the role of carbohydrates on GERD has not been well understood. 

Rice and wheat are the common food staples for humans worldwide. Besides starch, wheat also contains different proteins, such as gluten, albumin, globulin, and polypeptides [13]. Among them, gluten is the major protein component. In contrast, albumin, globulin, and glutelin are the major protein in rice [14]. Likewise, starch components in rice and wheat are also different. Rice and wheat are representative of a low and high FODMAPs diet, respectively. Rice has been proposed as a good carbohydrate source for patients with functional gastrointestinal disorders [15]. Studies showed that hydrogen gas, a colonic bacterial fermentation product, in breath samples after rice ingestion is not significantly increased from the fasting period. In contrast, wheat ingestion produced more breath hydrogen gas in healthy humans [16] and patients with IBS who had negative tests for celiac disease [17]. This suggests that wheat is not completely absorbed in the small intestine, and it may produce GI symptoms independent of gluten hypersensitivity. 

Therefore, this study aimed to evaluate the effects of low vs. high FODMAPs meals on typical GER symptoms using rice vs. wheat noodles, and the correlation between GERD symptoms and intestinal gas production in patients with overlapping GERD-IBS. We hypothesized that ingestion of wheat, a high FODMAP food, will increase colonic fermentation, intestinal gas, and SCFAs, leading to more TLESRs and GERD symptoms than rice, a low FODMAP food.

## 2. Materials and Methods

### 2.1. Study Subjects

Adult patients (aged 18–65 years old) who had typical GERD symptoms (bothersome heartburn and/or regurgitation) and non-constipation type IBS according to the Rome III criteria were recruited from the gastroenterology clinic in King Chulalongkorn Memorial Hospital, The Thai Red Cross Society, Bangkok, Thailand. A symptom questionnaire with a Bristol Stool Form Scale (BSFS) was used to exclude the IBS constipation-predominant type. We excluded patients with a history of allergies to the test meals; history of abdominal surgery, except for appendectomy and hemorrhoidectomy; pregnancy; major psychological disorders; and comorbid pulmonary conditions, such as chronic obstructive pulmonary disease (COPD). A serologic test for celiac disease (serum immunoglobulin A, anti-tissue transglutaminase; tTG antibody), and a specific skin prick test for gluten and wheat allergy (ALK Abello Pharm., Inc., Mississauga, ON, Canada) were performed. All participants needed to have stable medical treatment at least four weeks before the study enrollment; stop probiotics, antibiotics, prokinetics, laxatives, or medications that affect GI functions and symptoms during a 2-week run-in and the study period; and record their food diary for three days before the study. Patients who still had overall GI symptoms severity during the last week of a run-in period more than 5 of 10 cm of visual analog scale (VAS) were enrolled. 

### 2.2. Study Design

All subjects were randomly assigned by block randomization to the different test meals (rice noodles or wheat noodles), then crossover with a one-week washout period. This period was judged as a sufficient time for the meals in the previous period to have washed out, as the participants had a stool frequency of at least three times per week. After at least 8 h fasting, baseline GI symptom scores and BSFS were assessed. All subjects ingested a standard 250 g rice noodles or 250 g wheat noodles at 8.00 AM (breakfast) and noon (lunch). Exhaled breath hydrogen (H_2_) and methane (CH_4_) gas were acquired from all subjects at fasting and after breakfast every 15 min for 8 h. Although the rice and wheat noodles looked different, all patients were not informed about the major component of the noodles, and the term “FODMAPs” was not mentioned in the patient’s information sheet. The investigators who evaluated GI symptoms and measured the breath H_2_ and CH_4_ gas levels were blinded to the test meals. The postprandial GI symptoms, including typical GERD symptoms (heartburn, regurgitation), gas-related symptoms (bloating, belching, flatulence), satiety, nausea, abdominal pain, and stool urgency, were evaluated using 10 cm visual analog scales (VAS), in which 0 indicated no symptom and 10 indicated the most severe symptoms. All subjects gave their informed consent before they participated in the study. The study was conducted following the Declaration of Helsinki, and the protocol was approved by the Institutional Review Board of Faculty of Medicine, Chulalongkorn University (project identification code 027/56 and 678/62).

### 2.3. Interventional Meals

The study meals were made from 90-g-dry weight of rice or wheat noodles, and cooked as 250-g-cooked weight, as described in our previous study [17]. No vegetable or other fermentable ingredients, such as garlic or soy sauce, were added, to avoid intestinal gas production from other sources. In each study, participants took the test meal for breakfast and lunch. A glass of water (250 mL) was allowed with the test meals, and subjects were asked to finish their meal within 15 min. No food, drink, or medication were allowed during the study. We recorded the amount of food taken in every meal. According to the USDA Food Composition Database (https://ndb.nal.usda.gov/ndb/search/list (accessed on 30 November 2021)) [18], the total energy in each serving size for wheat and rice noodles in this study was 440 kcal and 450 kcal, respectively. The carbohydrate, protein, fat, and fiber contents in wheat and rice noodles serving were 42:32:14:2 g and 50:30:14:<1 g, respectively. The oligosaccharides (fructans and galacto-oligosaccharides) for a grain cutoff value of less than 0.3 g per serving was classified as a low FODMAPs diet, which was well tolerated and did not trigger symptoms in patients with IBS in clinical studies [19,20,21]. A study reported a serving size (cooked, 165 g) of wheat-based pasta containing 2.5 g fructans, which is considerably high in FODMAPs content [22]. In contrast, 100 g cooked rice noodles did not contain fructans and short-chain carbohydrates [23]. In this study, we used 250 g rice noodles, representing a low FODMAPs meal; and 250 g wheat noodles, representing a high FODMAPs meal.

### 2.4. Breath Tests

In the evening before the study day, we advised all subjects to avoid poorly absorbable carbohydrates affecting intestinal gas production on the study day. Patients ensured their good oral hygiene during the breath testing by brushing their teeth before taking the first breath sample [24]. The baseline gas sample was a fasting sample taken before breakfast, then every 15 min for 8 h. Each breath sample was collected using a 250 mL sample holding bag (Quintron Instrument Co., Inc., Milwaukee, WI, USA). We used a Quintron Microlyzer Model DP Plus (Quintron Instrument Co., Inc., Milwaukee, WI, USA) for measuring H_2_ and CH_4_, and reported in parts per million (ppm). 

### 2.5. Statistical Analysis

The primary outcome was the postprandial typical GERD symptoms (heartburn, regurgitation) severity scores compared between the test meals. Secondary outcomes were the gas-related symptoms (bloating, belching, flatulence) and other GI symptom scores after breakfast and lunch, and exhaled H_2_ and CH_4_ over an 8 h study comparing the interventional meals. The sample size was calculated to determine at least a 30% difference of GERD symptom severity score between rice and wheat [25] with 90% power at α = 0.05, and at least 20 subjects were needed.

A comparison of GI symptoms, H_2_, and CH_4_ gas levels between two groups were analyzed using the paired T-test and Wilcoxon sign rank test, depending on data distribution. A *p*-value of less than 0.05 was defined as statistical significance. Data were expressed as mean ± SD or median (interquartile range) as appropriate. The repeated measures analysis of variance was also performed to determine whether period, sequence, and carryover effects can arose in a crossover trial. The data were analyzed using SPSS software version 26.0 for Windows.

## 3. Results

Twenty-one patients (18F, age 50 ± 13 years, BMI 24.1 ± 4.3 kg/m^2^) with bothersome typical GERD symptoms overlapping non-constipation type IBS were included. The duration of GERD and IBS symptoms was 6 (5–12) and 8 (6–13) months, respectively. Baseline global symptoms severity (VAS 0–10) was 7.6 ± 1.5, with a typical GERD symptoms severity score of 6.1 ± 2.3 at the study enrollment. The median BSFS during the past month before study enrollment was 5(4–5), with a stool frequency of 7(6–10) times per week. Thirteen patients had serologic tests for celiac disease (serum immunoglobulin A, anti-tissue transglutaminase; tTG antibody), and a specific skin prick test for gluten and wheat allergy, and all the results were negative. Nineteen patients underwent upper endoscopy after GERD onset, and all of them had no reflux esophagitis. The other two patients did not undergo upper endoscopy as they were under 50 years and had no alarm features. Three-day food diaries before the study day showed comparable food items between arms. The fasting GI symptom scores in the morning before each study meal ingestion were not significantly different compared between test meals (*p* > 0.05) (Table 1). 

### 3.1. Effects of Wheat vs. Rice Ingestion on Typical GERD Symptoms

**Figure 1:**
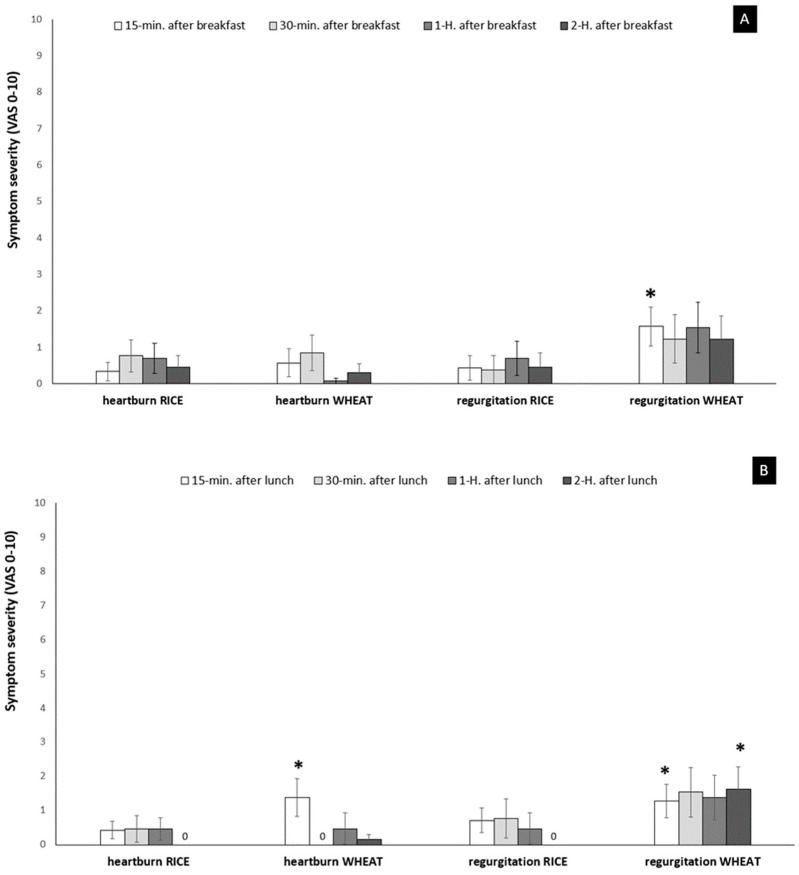
The typical gastroesophageal reflux disease (GERD) symptoms scores at postprandial period comparing between wheat and rice noodles ingestion. (**A**) shows symptoms after breakfast, and (**B**) shows symptoms after lunch. Heartburn and regurgitation were evaluated by visual analog scale (VAS) 0–10. * *p* < 0.05 wheat noodles vs. rice noodles. Data expressed as mean and SEM.

All patients finished the assigned wheat and rice noodles at a similar amount, and completed the studies without serious adverse events. Regarding the symptoms after breakfast, regurgitation severity after the wheat noodles arm was significantly higher than rice noodles only at 15 min (*p* < 0.05). Then, the symptom gradually decreased, and was not significantly different between wheat and rice noodles after that. Heartburn symptoms 2 h after wheat noodles for breakfast were not significantly different from rice noodles (*p* > 0.05). In contrast, at 15 min after lunch, heartburn and regurgitation severity in the wheat noodles arm were significantly higher than the rice noodles arm (*p* < 0.05). Also, at 2 h after lunch, regurgitation severity in the wheat noodles arm was significantly higher than the rice noodles arm (*p* < 0.05) (Figure 1A,B).

### 3.2. Effects of Wheat vs. Rice Ingestion on Other GI Symptoms

**Figure 2:**
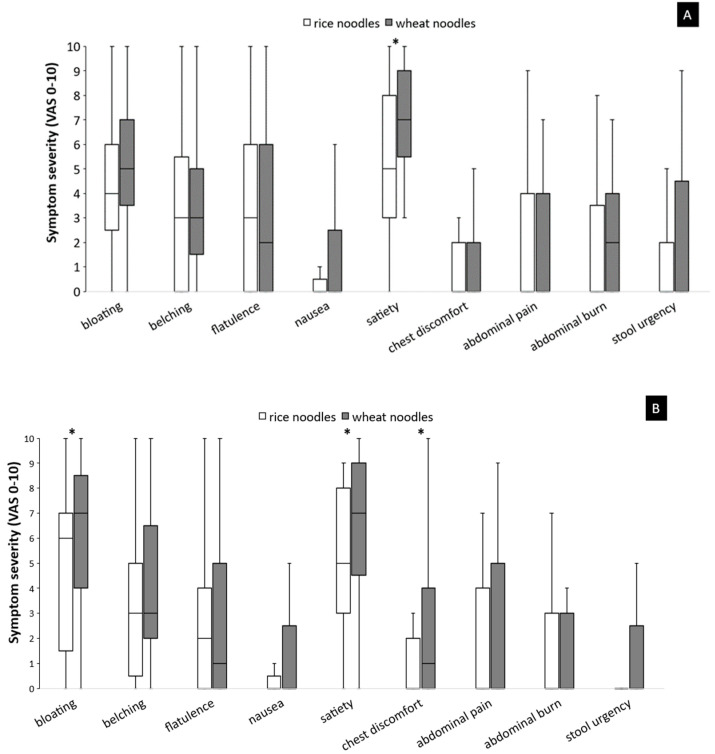
The maximal gastrointestinal symptom scores during 4 h after breakfast (**A**) and lunch (**B**), comparing between wheat and rice noodles ingestion. Symptoms were evaluated by visual analog scale (VAS) 0–10. A box and whisker plot represents the median, interquartile range, minimal, and maximal values of symptom severity. * *p* < 0.05 wheat noodles vs. rice noodles.

During 2 h after breakfast, wheat noodles ingestion was significantly associated with higher maximal satiety symptom scores than rice noodles ingestion (*p* < 0.05), whereas bloating, belching, flatulence, nausea, chest discomfort, abdominal pain, abdominal burn, and stool urgency severity scores were not significantly different compared to rice noodles (*p* > 0.05). In contrast, wheat noodles ingestion significantly aggravated more severe bloating, satiety, and chest discomfort than rice noodles ingestion during 2 h after lunch (*p* < 0.05). Belching, flatulence, nausea, abdominal pain, abdominal burn, and stool urgency severity scores were not significantly different after the wheat and rice test meals. Wheat noodles for lunch were associated with more 2 h postprandial bloating symptoms than wheat noodles for breakfast (*p* < 0.05) (Figure 2).

### 3.3. H_2_ and CH_4_ Production after Rice vs. Wheat Ingestion

**Figure 3:**
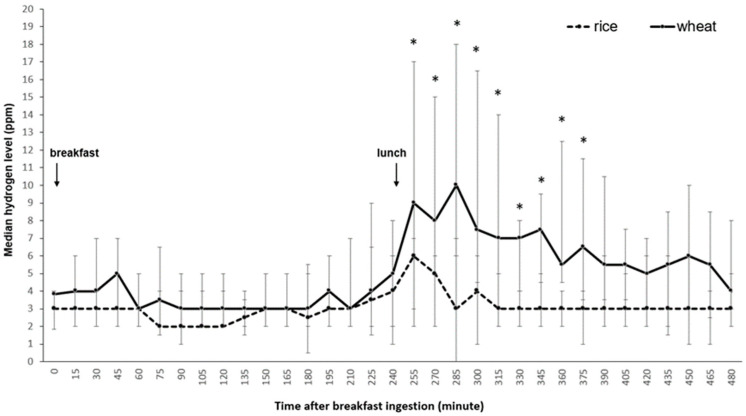
Exhaled hydrogen gas concentrations after ingestion of different test meals. * *p* < 0.05 wheat noodles vs. rice noodles.

The area under the curve (AUC) of H_2_ and CH_4_ concentration over 8 h after wheat noodles ingestion were significantly greater than the levels after rice noodles ingestion (wheat vs. rice; AUC: H_2_ = 2925(1710–5334) vs. 1523(944–2446) ppm-min; CH_4_ = 833(581–1628) ppm-min, *p* < 0.001). During 4 h after lunch, the AUC of H_2_ and CH_4_ concentration in the wheat study arm were significantly higher than the rice study arm (AUC: H_2_ = 1208(300–2145) vs. 600(278–840) ppm-min; CH_4_ = 390(296–1050) vs. 330(214–566) ppm-min, *p* < 0.05). In the wheat noodles arm, the AUC of H_2_ and CH_4_ concentrations after lunch were significantly higher than the concentrations after breakfast (*p* < 0.05), but not rice (*p* > 0.05). This effect was demonstrated immediately after lunchtime (Figure 3). During 4 h after breakfast, the AUC of H_2_ and CH_4_ concentration were not significantly different between wheat and rice noodles ingestion (AUC: H_2_ = 533(266–999) vs. 485(248–739) ppm-min; CH_4_ = 350(243–494) ppm-min, *p* > 0.05). Also, H_2_ and CH_4_ concentration levels at every time point during this period were similar.

### 3.4. The Correlation between Postprandial Symptoms and H_2_ and CH_4_ Production

The maximum regurgitation symptom was developed at the first 15 min after lunch in nearly all (19/21) patients. Thus, we correlated the regurgitation severity scores with H_2_ and CH_4_ concentrations during 15 min after lunch. The regurgitation severity score at 15 min after wheat noodles for lunch significantly correlated with the area under the curve of exhaled H_2_ concentration during 15 min after lunch (r = 0.56, *p* = 0.009) and the area under the curve of exhaled CH_4_ concentration during 15 min after lunch (r = 0.55, *p* = 0.009). 

In the analysis of variance, there was no indication of a sequence effect, a period effect, or a carryover effect on the GI symptoms, and gas production (*p* > 0.05)

## 4. Discussion

This study demonstrated wheat noodles, a high FODMAPs meal, were significantly associated with higher postprandial heartburn and regurgitation than rice noodles, a low FODMAPs meal, in patients with GERD overlapping non-constipation type IBS. This effect was clearly observed after lunch. In addition, wheat produced higher exhaled H_2_ and CH_4_ concentrations than rice noodles, demonstrated immediately after lunchtime onward, but not after breakfast. Moreover, after lunch, the amount of H_2_ and CH_4_ production with wheat noodles significantly correlated with postprandial GERD symptom severity. This finding suggests that intestinal gas production, or colonic fermentation of incomplete carbohydrate absorption might play roles in the postprandial GERD symptoms after lunch. This study also demonstrated that regurgitation symptom scores, but not heartburn scores, after wheat noodles for breakfast were significantly higher than rice noodles, independent of intestinal gas production. All thirteen patients who underwent serologic tests for celiac disease, and specific skin prick tests for gluten and wheat allergy had negative results. Wheat noodles still have different components than rice noodles, such as gluten [13,14], and may trigger more regurgitation symptoms without increased intestinal gas in the morning. Furthermore, this effect was augmented by gas production in the afternoon, as both heartburn and regurgitation symptoms scores after wheat noodles were significantly higher than rice noodles. This finding suggests that mechanisms other than colonic fermentation might also play a role in the pathogenesis of wheat-induced regurgitation symptoms. 

The rising rate of exhaled intestinal gas being the highest at the first 15 min after wheat lunch suggests a clearance of incomplete absorbed carbohydrates from the ileum into the large bowel stimulated by lunch ingestion. Studies showed that the bacterial fermentation products in the colon not only stimulate colonic propagated contraction, and accelerate colonic transit [3,4], but also decrease proximal gastric tone in a dose-dependent manner [5], and induce transient lower esophageal sphincter relaxations (TLESRs) [6], and TLESR-associated acid reflux episodes in healthy individuals [6]. The maximal difference in regurgitation and heartburn severity scores between wheat and rice in this study occurred at the first 15 min after lunch. As a randomized crossover design, wheat-induced regurgitation and heartburn symptoms after lunch likely link to TLESRs via colonic fermentation. 

The participants in our study also had a more increased regurgitation after breakfast with wheat noodles than rice noodles, which were not associated with the intestinal gas production amount. After breakfast, the difference in GERD symptoms was only regurgitation, but not heartburn, and symptoms were rapidly improved rather than prolonged, and associated with other gas-related symptoms in the afternoon. The mechanisms independent of colonic fermentation that explain wheat-induced regurgitation symptoms in the morning remain elusive. One hypothesis is the impact of different diets on reflux perception. A recent randomized study comparing between a 4-week low FODMAPs diet (< 3 g/d) and a diet following usual dietary advice (low-fat diet; avoid alcohol, caffeine, and overeating) in the PPI-refractory GERD patients who had normal total acid exposure time ((AET), (median AET 1.1% (0.2–2.6)) demonstrated a similar GERD symptoms improvement [26]. Almost all patients in both groups displayed unchanged pH-impedance parameters (reflux number, acid exposure, bolus exposure time) at the end of the diet. In contrast, the number of patients with positive symptom association numerically decreased. So, different food components or dietary modifications might affect GERD symptoms perception in patients with PPI-refractory GERD. A physiologic study in healthy subjects showed that food could activate gastric accommodation, an underlying mechanism of GER [27], since it is in the mouth without swallowing (oropharyngeal phase) to the duodenal phase [28]. A study in NERD patients from our center showed that capsaicin, a TRPV1 receptor agonist, enhanced gastric accommodation more than placebo in GERD patients, and this effect appeared at 20 min postprandial period [29]. Whether different chemical stimuli from wheat, rice, or their components may enhance gastric accommodation in GERD patients differently is not known. A pre-clinical study showed that hexane extract of wheat flour could activate capsaicin (TRPV1) receptors, but do not have a pungency effect [30]. This finding may explain why only regurgitation, but not heartburn, symptoms were triggered after wheat ingestion in the morning in the present study. 

Most starch in the western diet usually comes from refined wheat grains. The Monash University FODMAPs Diet Application^®^ classified wheat as a high FODMAPs food, and rice as a low FODMAPs food. This study uses 250 g rice noodles, representing a low FODMAPs meal; and 250 g wheat noodles, representing a high FODMAPs meal. Although, this study suggests the possible role of a low FODMAPs diet as a non-pharmacologic treatment for GERD with overlapping IBS patients. It is impossible to exclude the effect of gluten or other components in wheat noodles on GI symptoms and intestinal gas production. Previous studies in healthy volunteers showed that gluten-containing wheat meals produce a significantly higher cumulative breath H_2_ excretion than gluten-free wheat meals and gluten-free wheat meals with added gluten [31,32]. A previous study from our group also showed that mung bean noodles, considered as gluten-free noodles, produce intestinal gas comparable to rice noodles, although mung beans are classified as a high FODMAPs food [17]. It is possible that the procedures for gluten extraction from the flour altered the carbohydrates, making them more absorbable. Further studies are needed to elucidate whether FODMAPs, gluten, or other components in wheat induce GERD symptoms. 

The effectiveness of a gluten-free diet and low FODMAPs for GERD management has been reported [26,33,34,35]. A prospective study in untreated celiac disease patients showed that GERD is six-fold more common than healthy individuals. After treatment with a gluten-free diet, GERD symptoms rapidly improved during the first three months, and there was a persistent improvement, despite PPIs being discontinued [33]. Thus, the mechanism of GERD in celiac disease patients might be different from GERD in the general population, and diet might play an important role. A linkage between GERD symptoms and gluten/wheat ingestion has been reported in non-celiac disease patients. A study among 498 patients without celiac disease who were referred to a digestive endoscopy showed that 20% of them reported GERD symptoms after gluten/wheat ingestion at least once per week, and symptom disappearance on a gluten/wheat-free diet. The GERD prevalence in patients with self-reported wheat sensitivity was more than in the control group [36]. The beneficial effect of a low FODMAPs diet other than a gluten-free diet on GERD has yet to be explored.

Limitations of our study are: (1) We did not perform an esophageal pH study in all patients. Although almost all the participants had no esophagitis, the study results could not be generalized to all GERD phenotypes and GERD patients without IBS. Future studies on GERD patients with esophagitis or significant esophageal acid exposure may provide different results. (2) Although the enrolled patients reported bothersome heartburn and/or regurgitation during the last week of a run-in period, the postprandial GERD symptoms severity after tested meals was low. The tested food may be different from patients’ usual food. It may not induce GERD symptoms as severe as the patients’ previous experience, even though the difference in symptom severity was statistically significant between study arms. (3) It is difficult to make two different study meals identical looking. We tried to minimize these limitations by avoiding informing the patients about the carbohydrates sources and FODMAPs. Previous experience of the patients with food might cause an expected symptoms bias. However, we demonstrated the impact not only on the patient symptoms, but on objective parameters, which are the intestinal gases and blinding the study meals to the assessors. (4) Other high FODMAP starch might cause different effects from wheat noodles. A longer period of ingestion might also cause different effects from the two meals ingestion in this study. So, this needs to be evaluated before a universal recommendation of low FODMAPs diets in overlapping GERD-IBS to treat GERD and upper GI symptoms. 

## 5. Conclusions

This study demonstrated that wheat noodles, a high FODMAP meal, produce more typical GERD symptom scores postprandially, especially early or the first 15 min after lunch, than rice noodles, a low FODMAPs meal. The effect of wheat noodles on GERD symptoms is associated with increased intestinal gas production after lunch, confirming the linkage between GERD symptoms and colonic fermentation. Other mechanisms of wheat-induced GERD symptoms after breakfast independent of gas production must be further explored. This study suggests that rice is a better source of carbohydrates for patients with overlapping GERD and non-constipation type IBS than wheat, and provides insight into the role of a FODMAPs dietary modification for treating these patients. 

## Figures and Tables

**Table 1:** Fasting gastrointestinal symptom scores in the morning before each study meal ingestion.

	Wheat Noodles (*n* = 21)	Rice Noodles (*n* = 21)
**GERD symptoms (Visual analog scale 0–10)**		
Heartburn	0(0–0)	0(0–0)
Regurgitation	1(0–2.5)	0(0–0)
**Other GI symptoms (Visual analog scale 0–10)**		
Bloating	3(0–5.5)	3(0–3.5)
Belching	2(0–6)	2(0–3.5)
Flatulence	2(0–6)	1(0–4)
Nausea	0(0–1.5)	0(0–1)
Satiety	5(3.5–5.5)	5(2–6)
Chest discomfort	0(0–0)	0(0–0)
Abdominal pain	0(0–2.5)	0(0–3)
Abdominal burn	0(0–2.5)	0(0–2.5)
Stool urgency	0(0–0)	0(0–0)

Data expressed as median (interquartile ranges; *p* > 0.05, wheat noodles vs. rice noodle for all symptoms.
